# Influence of somatic cell count, body condition and lameness on follicular growth and ovulation in dairy cows

**DOI:** 10.1016/j.theriogenology.2008.10.001

**Published:** 2009-03-15

**Authors:** M.J. Morris, S.L. Walker, D.N. Jones, J.E. Routly, R.F. Smith, H. Dobson

**Affiliations:** Department of Veterinary Clinical Science, Faculty of Veterinary Science, University of Liverpool, Leahurst, Neston, Wirral CH64 7TE, UK

**Keywords:** Stress, Dairy cattle, Follicle, Ovulation, Subfertility

## Abstract

The objective of this study was to investigate the effect of somatic cell count (SCC), body condition score (BCS) or lameness score on ovarian follicular growth and ovulation in dairy cows. Seventy four animals 30–80 days post-partum were monitored for all three conditions before synchronization of ovarian follicular phases by administration of gonadotrophin releasing hormone (GnRH) followed seven days later with prostaglandin F2alpha (PG). Ultrasonography of both ovaries twice daily throughout the follicular phase revealed that fewer animals with combined high SCC and lameness (4/9) ovulated compared to healthy animals (19/21; *P* *=* 0.006) or animals with only high SCC (11/11; *P* = 0.004) or only lameness (21/27; *P* = 0.06). Overall, regardless of the presence of other concurrent conditions, fewer lame cows ovulated than Non Lame animals (30/42 and 30/32; *P* *=* 0.015). Mean follicular growth and maximum follicular diameter were unaffected by any of the three conditions. However, dominant follicle growth and maximum diameter were greater in the 60 animals that ovulated compared to the 14 that did not; 1.83 ± 0.16 versus 0.96 ± 0.26 mm/day (*P* = 0.014) and 19.4 ± 0.4 versus 16.4 ± 1.2 mm (*P* *=* 0.003), respectively. In conclusion, lameness reduced the proportion of cows that ovulated and the synergistic effect of high SCC and lameness reduced that proportion further. However, follicular growth and maximum follicular diameter were unaffected by high SCC, low BCS or lameness.

## 1 Introduction

The stress of various production ‘diseases’ has an adverse effect on dairy cow fertility with clinical mastitis [1], low body condition score [2] and lameness [3–5] being especially deleterious. Subclinical mastitis is also an economically important disease stressor [6]; and this prompted the investigation of high somatic cell count (SCC) animals rather than clinical cases of mastitis. It has been suggested that stressors, such as these clinical conditions, disrupt gonadotropin support required for appropriate follicular growth and ovulation, with consequent effects on fertility [7,8].

The use of frequent real time trans-rectal ultrasonography allows close monitoring of ovarian activity, especially enabling the precise measurement of follicular diameter, mean growth rate and time of ovulation, without itself influencing the results [9]. Using this technique, heat stress in cattle has been shown to result in slower follicular growth concomitant with atypical steroidogenic profiles and smaller dominant follicles [10,11]. Similarly, cows in nutritionally-induced anoestrus have smaller dominant follicles [12].

Stressors often occur in combination; therefore, the present study investigated the role of the common production conditions (high SCC, low body condition and/or lameness), severally or singly, on follicular growth and ovulation in dairy cows. The cumulative effect of diseases may synergize, pushing the affected animals “over the edge” with respect to follicular growth and the ability to ovulate. Furthermore, to ensure that the data are meaningful to the dairy industry, the study was carried out on commercial farms with as little disruption as possible to normal management practices.

We began with the hypothesis that dairy cows with the most common production conditions (high SCC, low body condition and/or lameness) have smaller follicles growing at a slower rate, ultimately resulting in a lower proportion of ovulations than in healthy counterparts living in the same environment.

## 2 Materials and methods

The study was conducted from May to November in three successive years on two commercial farms with 200 and 130 cows, respectively. There were 49, 0, 19 and 0, 20, 2 cows in each year on each farm, respectively. The 90 multiparous lactating Holstein cows enrolled in the study had an average milk yield of 8800 kg per lactation with milking (including study cows) starting at 6 h and 16 h each day. Animals were enrolled from the whole herd as those without any confounding clinical conditions except high SCC, low body condition and/or lameness. Cows randomly entered the study only once between 30 – 80 days post partum and, at any one time, no more than 12 cows were monitored. During the study periods, animals on one farm were out at grass with a supplementary Total Mixed Ration (TMR) fed indoors for 1 h immediately after each milking. Pastures were of seasonal ryegrass, Italian ryegrass and white clover. Cows on the second farm were kept inside throughout and fed TMR (including haylage and alkalage) *ad libitum*. Cows had been routinely hoof trimmed at the end of the previous lactation. The study was performed under a UK Home Office license for work on living animals and with the approval of the University of Liverpool Ethical Review Process.

Individual cow somatic cell counts (SCCs) from a pooled milk sample from all quarters of the udder were measured every 4–6 weeks by commercial companies employed by the individual farms (National Milk Records Plc, Chippenham, UK; or the Cattle Information Service, Watford, UK). Animals with overt clinical mastitis (presence of clots or watery milk, with or without inflamed udder) were excluded from the study. Cell counts of the study cows immediately prior to oestrous cycle synchronization were used to define the prevailing SCC status of the cow. A cell count <100,000 cells/ml was classified as low SCC and a count ≥100,000 cells/ml was classified as high [13].

Body condition scores (BCS) were determined using a 1–5 system incorporating 0.5 scores [14]. Animals with a mean BCS < 1.5 were classified as low BCS and those with a mean score ≥1.5 or more were classified as moderate BCS. The cows were also scored for lameness at the same time as BCS, using a standardized 1–5 system [15]. Both were performed weekly from 3 weeks before oestrous cycle synchronization for a total of 5 weeks and the mean scores over the time period were calculated. Throughout the study, the lameness score of 95% of individuals was the same, or one out of five assessments was within 1 score. Animals with a mean score <1.5 were classified as Non Lame and those with a mean score of ≥1.5 or more were classified as Lame.

### 2.1 Oestrous cycle synchronization

Recent studies have indicated that lame cows inherently have lower progesterone concentrations in the luteal phase prior to expressing oestrus [8]. Thus, to facilitate frequent ultrasound examinations from a sufficient number of cows, ovarian follicular phases were synchronized using a hormonal regime that did not involve administration of exogenous progesterone to avoid disrupting the endogenous progesterone milieu. Thus, cows received 100 μg buserelin (GnRH; 2.5 ml Receptal^®^; Intervet, Milton Keynes, UK) at morning milking followed seven days later by 500 μg cloprostenol (PG; 2 ml Estrumate^®^; Schering-Plough, Uxbridge, UK).

### 2.2 Milk sampling

Milk samples were taken on alternate days for 3 weeks prior to GnRH administration and then daily until 2 days after PG injection to determine the response to the synchronization regime. All samples were taken immediately before milking and promptly stored at −20 °C without preservative.

### 2.3 Ultrasonography

The ovaries of all animals were scanned twice daily *per rectum* with a Concept/MCV Veterinary Ultrasound Scanner using a 7.5 MHz linear array probe (Dynamic Imaging, Livingstone, Scotland) from PG administration until ovulation or until the appearance of a new follicular wave. Follicles (F) were identified as non-echogenic structures with a defined border between the follicular wall and antrum. Corpora lutea (CL) were identified as grainy echogenic structures with a distinct demarcation from the less echogenic normal ovarian stroma [16]. Diameters were calculated as the average of two perpendicular measurements. Dominant follicles were defined as those that achieved an internal diameter ≥ 10 mm in the absence of other actively growing follicles [17,18]. Maximum diameter of the dominant follicle was defined as the diameter just before ovulation (mean = Day 4.5 ± 0.2 after PG), or the diameter on Day 4.5 in those animals that failed to ovulate. Follicular growth was determined as the mean positive change in follicular diameter between time of PG administration and last ultrasound before ovulation. Ovulation was considered to have occurred when a follicle >10 mm was absent at the following ultrasonography session 12 h later.

### 2.4 Hormone assays

Progesterone, analyzed as ‘pregnane metabolites’, was measured in 50 μl whole milk samples using an established EIA assay [8]. Samples were compared with a standard preparation of progesterone obtained from Sigma–Aldrich, Poole, UK (Cat. # P0130). The minimum detectable amount was 0.015 ng/ml; and the intra- and inter-assay coefficients of variation were 8.3% and 14%, respectively.

### 2.5 Statistical analysis

All data were analyzed using Minitab (Version 14, Minitab Inc. Pennsylvania, USA) with data expressed as mean ± SEM or proportions where appropriate. Differences were considered significant when *P* *<* 0.05 and reported as a tendency when 0.05 < *P* < 0.10. Associations between ovulation and conditions were examined by *χ*-square analysis. General linear model analysis of variance (GLM ANOVA) was used to analyze the effect of SCC, lameness and body condition scores on follicular growth rate, maximum follicular diameter (with farm and year included initially as explanatory variables, but later excluded as no influence was detected). Factors affecting the time to ovulation were identified using regression with life data, right censored at 7.5 days for animals that did not ovulate.

## 3 Results

Throughout the study, 16 animals had progesterone concentrations below baseline (0.17 ng/ml; mean lowest value derived from the mean plus twice the SD progesterone concentration during the follicular phase of healthy cows; Walker et al. [8]). These animals had not responded to ovarian synchronization and were removed from the study. All these animals with continuously low progesterone were Lame and none ovulated resulting in a synchronization failure rate of 28% for all Lame cows (and 18% failure for all cows). A total of 74 animals did respond to synchronization and were used for further statistical analysis.

**Fig. 1:**
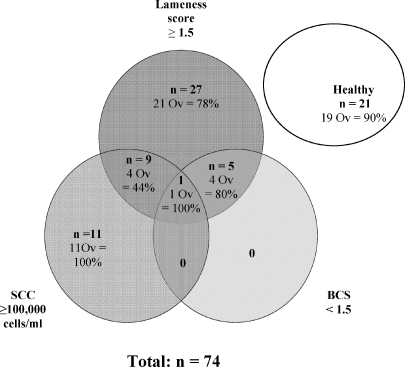
Numbers of animals (**bold type**) that were healthy or had high SCC, low BCS, lameness or a combination of conditions. Also shown are numbers and percentages within each subgroup that ovulated (Ov).

Fifteen cows had more than one concurrent condition (mastitis, low body condition and/or lameness); the group distributions are shown in Fig. 1. At the time of PG injection, each cow regardless of group had a follicle of at least 10 mm diameter that grew to a maximum of 14–33 mm. The mean follicular growth rate, maximum follicular diameter and time to ovulation were similar in all groups (*P* > 0.1, GLM ANOVA; Table 1).

Fig. 1 also reveals that the proportions of animals that ovulated were not different between the healthy group (19/21) and the high SCC only (11/11; *P* = 0.909, *χ*-Sq = 0.013) or Lame only groups (21/27; *P* = 0.242, *χ*-Sq = 1.371). However, fewer Lame animals with high SCC ovulated compared to healthy animals (4/9 versus 19/21 *P* *=* 0.006; *χ* -square = 7.462). Lame + low BCS cows and healthy animals were not compared due to the low numbers of low BCS animals.

Fewer cows with concurrent high SCC and Lameness (4/9) ovulated compared to those animals with only high SCC (11/11; *P* = 0.004, *χ*-Sq = 8.15); and there tended to be fewer cows with both conditions that ovulated compared to cows that were only Lame (21/27; *P* = 0.06 *χ*-Sq = 3.54).

There were no differences between the proportion of cows ovulating in the Lame + low BCS group (4/5) compared with the Lame only cows (21/27; *P* = 0.91; *χ*-square = 0.012); or the Lame + high SCC cows (4/9; *P* = 0.20; *χ*-square = 1.659).

Overall analysis of the individual disease conditions (regardless of the presence of other conditions), revealed that the proportions ovulating were similar between animals with high or low SCC (16/21 and 44/53; *P* *=* 0.50; *χ*-square = 0.46); and low or moderate BCS (5/6 and 55/68; *P* *=* 0.88, *χ*-square = 0.02). However, fewer Lame compared to Non Lame animals ovulated (30/42 and 30/32; *P* *=* 0.015; *χ*-Sq = 5.89).

The dominant follicle grew faster in animals that ovulated compared to those that did not (1.83 ± 0.16, (*n* = 60) versus 0.96 ± 0.26 mm/day (*n* = 14); *P* = 0.014). The maximum diameter of the dominant follicle was also larger in the ovulating cows (19.42 ± 0.39 versus 16.43 ± 1.22 mm; *P* *=* 0.003).

## 4 Discussion

Overall, the proportion of animals that ovulated was depressed only in the Lame animals. However, in the presence of both Lameness and high SCC, the proportion of ovulating animals was lower than with each condition alone. Dominant follicles grew at the same rate to the same maximum diameter and ovulated at the same time as healthy animals.

Of all the cows subjected to the synchronization regime, a proportion (18%) did not respond, with progesterone concentrations remaining at or below baseline for the duration of the study. This failure rate is comparable with that found by others (20–30%; [19,20] and could be associated with failure of commencement of luteal activity (CLA). Spontaneous CLA is delayed in 15% of post partum cows and occurs 18 days late in Lame animals, increasing intervals from calving to first AI, and to conception [21,22].

Per-rectum ultrasonographic measurements of both follicular growth and maximum diameter were similar to other studies in postpartum cows [23–25], and concurred with results after experimentally induced mastitis [26]. Nevertheless, the present study is the first to show that ovulation is detrimentally affected. The lack of association of high SCC, low body condition and/or lameness with growth rate of dominant follicles and maximum diameter (contrary to our initial hypothesis) suggests different mechanisms are involved compared to heat stress, whereby follicles grow more slowly and achieve smaller maximum diameters [10,11,27]. Low dietary Dry Matter intake also resulted in smaller follicles reported in other studies [28,29]. As body condition was not reported in those studies, it is not clear how this relates to long-term low body condition score.

The lower proportion of ovulating lame cows explains the decreased fertility seen in various epidemiological field studies [3–5,30]. The sub fertility associated with high SCC [31] does not appear to be due to the same mechanism, as high SCC did not influence the proportion of cows ovulating in the present study. However, the two conditions together had a more profound negative effect on ovulation suggesting that the stressful effects were synergistic. On the two farms in the present study, there was a low incidence of low BCS, reducing the power of analysis. We have been unable to find other data in the literature linking the incidence of ovulation with low BCS although dietary restriction lowers the proportion of animals ovulating [29], and decreasing BCS reduces the conception rate to first insemination [32]. Furthermore, a reduction in BCS increases the interval from calving to first ovulation [2]. However, the present study was carried out over a relatively short period of time, preventing detection of changes in BCS.

In ovulating animals, irrespective of conditions, rates of growth and maximum follicular diameters were greater than in non-ovulating animals agreeing with previous studies [2,33].

In conclusion, we have investigated three common conditions, (high SCC, low BCS and lameness) that reduce fertility in the dairy cow. The mechanisms involved do not entail changes in follicular growth rate nor maximum follicle diameter. The current study supports the hypothesis that lame cows are less likely to ovulate; although the same is not true for animals with either high SCC alone or low BCS alone. Nevertheless, we have shown for the first time a synergistic effect of lameness and high SCC that further reduces the likelihood of ovulation. The mechanism by which this occurs requires further investigation.

## Figures and Tables

**Table 1:** Mean ± SEM follicular parameters, % cows ovulating and time to ovulation in cows with different conditions.

Follicular parameters	Healthy *n* = 21	High SCCs only *n* = 11	Lame only *n* = 27	Lame and high SCCs *n* = 9	Lame and low BCS *n* = 5
Mean follicular growth rate (mm/day)	1.5 ± 0.2	2.3 ± 0.5	1.8 ± 0.2	1.1 ± 0.5	1.3 ± 0.5
Max. follicular diameter (mm)	19.1 ± 0.6	19.2 ± 0.9	19.4 ± 0.5	16.0 ± 1.8	19.2 ± 2.9
Number of cows ovulating (%)	19 (90%)	11 (100%)	21 (78%)	4 (44%)	4 (80%)
Days to ovulation after PG	4.5 ± 0.2	4.4 ± 0.4	4.0 ± 0.1	4.3 ± 0.2	4.4 ± 0.7

There were no cows with low BCS only, or low BCS combined with high SCCs. One animal had all three conditions (data not shown).

## References

[1] Schrick F.N., Hockett M.E., Saxton A.M., Lewis M.J., Dowlen H.H., Oliver S.P. (2001). Influence of subclinical mastitis during early lactation on reproductive parameters. J Dairy Sci.

[2] Beam S.W., Butler W.R. (1999). Effects of energy balance on follicular development and first ovulation in postpartum dairy cows. J Reprod Fertil Suppl.

[3] Lucey S., Rowlands G.J., Russell A.M. (1986). The association between lameness and fertility in dairy cows. Vet Rec.

[4] Collick D.W., Ward W.R., Dobson H. (1989). Associations between types of lameness and fertility. Vet Rec.

[5] Melendez P., Bartolome J., Archbald L.F., Donovan A. (2003). The association between lameness, ovarian cysts and fertility in lactating dairy cows. Theriogenology.

[6] Huijps K., Lam T.J., Hogeveen H. (2008). Costs of mastitis: facts and perception. J Dairy Res.

[7] Dobson H., Smith R.F. (2000). What is stress, and how does it affect reproduction?. Anim Reprod Sci.

[8] Walker S.L., Smith R.F., Jones D.N., Routly J.E., Dobson H. (2008). Chronic stress, hormone profiles and estrus intensity in dairy cattle. Horm Behav.

[9] Roelofs J.B., Bouwman E.G., Dieleman S.J., Van Eerdenburg F.J.C.M., Kaal-Lansbergen L.M.T.E., Soede N.M. (2004). Influence of repeated rectal ultrasound examinations on hormone profiles and behaviour around oestrus and ovulation in dairy cattle. Theriogenology.

[10] Badinga L., Thatcher W.W., Diaz T., Drost M., Wolfenson D. (1993). Effect of environmental heat stress on follicular development and steroidogenesis in lactating Holstein cows. Theriogenology.

[11] Wilson S.J., Marion R.S., Spain J.N., Spiers D.E., Keisler D.H., Lucy M.C. (1998). Effects of controlled heat stress on ovarian function of dairy cattle. 1. Lactating cows. J Dairy Sci.

[12] Rhodes F.M., Entwistle K.W., Kinder J.E. (1996). Changes in ovarian function and gonadotropin secretion preceding the onset of nutritionally induced anestrus in Bos indicus heifers. Biol Repro.

[13] Dohoo I.R., Morris R.S. (1993). Somatic cell count patterns in Prince Edward Island dairy herds. Prev Vet Med.

[14] Chamberlain A.T., Wilkinson J.M. (1996). Feeding the dairy cow.

[15] Sprecher D.J., Hostetler D.E., Kaneene J.B. (1997). A lameness scoring system that uses posture and gait to predict dairy cattle reproductive performance. Theriogenology.

[16] Pierson R.A., Ginther O.J. (1984). Ultrasonography of the bovine ovary. Theriogenology.

[17] Dobson H., Ribadu A.Y., Noble K.M., Tebble J.E., Ward W.R. (2000). Ultrasonography and hormone profiles of adrenocorticotrophic hormone (ACTH)-induced persistent ovarian follicles (cysts) in cattle. J Reprod Fertil.

[18] Imwalle D.B., Fernandez D.L., Schillo K.K. (2002). Melengestrol acetate blocks the preovulatory surge of luteinizing hormone, the expression of behavioral estrus, and ovulation in beef heifers. J Anim Sci.

[19] Thatcher W.W., Macmillan K.L., Hansen P.J., Drost M. (1989). Concepts for regulation of corpus luteum function by the conceptus and ovarian follicles to improve fertility. Theriogenology.

[20] Twagiramungu H., Guilbault L.A., Proulx J., Villeneuve P., Dufour J.J. (1992). Influence of an agonist of gonadotropin-releasing hormone (buserelin) on estrus synchronization and fertility in beef cows. J Anim Sci.

[21] Petersson K.J., Gustafsson H., Strandberg E., Berglund B. (2006). Atypical progesterone profiles and fertility in Swedish dairy cows. J Dairy Sci.

[22] Petersson K.J., Strandberg E., Gustafsson H., Berglund B. (2006). Environmental effects on progesterone profile measures of dairy cow fertility. Anim Reprod Sci.

[23] Ginther O.J., Kastelic J.P., Knopf L. (1989). Composition and characteristics of follicular waves during the bovine estrous cycle. Anim Reprod Sci.

[24] Mann G.E., Robinson R.S., Hunter M.G. (2007). Corpus luteum size and function following single and double ovulations in non-lactating dairy cows. Theriogenology.

[25] Sunderland S.J., Crowe M.A., Boland M.P., Roche J.F., Ireland J.J. (1994). Selection, dominance and atresia of follicles during the oestrous cycle of heifers. J Reprod Fertil.

[26] Hockett M.E., Almeida R.A., Rohrbach N.R., Oliver S.P., Dowlen H.H., Schrick F.N. (2005). Effects of induced clinical mastitis during preovulation on endocrine and follicular function. J Dairy Sci.

[27] Roth Z., Meidan R., Braw-Tal R., Wolfenson D. (2000). Immediate and delayed effects of heat stress on follicular development and its association with plasma FSH and inhibin concentration in cows. J Reprod Fertil.

[28] Murphy M.G., Enright W.J., Crowe M.A., McConnell K., Spicer L.J., Boland M.P. (1991). Effect of dietary intake on pattern of growth of dominant follicles during the oestrous cycle in beef heifers. J Reprod Fertil.

[29] Diskin M.G., Mackey D.R., Roche J.F., Sreenan J.M. (2003). Effects of nutrition and metabolic status on circulating hormones and ovarian follicle development in cattle. Anim Reprod Sci.

[30] Fourichon C., Seegers H., Malher X. (2000). Effect of disease on reproduction in the dairy cow: a meta-analysis. Theriogenology.

[31] Santos J.E.P., Cerri R.L.A., Ballou M.A., Higginbotham G.E., Kirk J.H. (2004). Effect of timing of first clinical mastitis occurrence on lactational and reproductive performance of Holstein dairy cows. Anim Reprod Sci.

[32] Bourchier C.P., Hutchinson J.M., Benson T.A. (1987). The relationship between milk yield, body condition and reproductive performance in high yielding dairy cows. Anim Prod.

[33] Maquivar M., Verduzco A., Galina C.S., Pulido A., Rojas S., Forster K. (2007). Relationship among follicular growth, oestrus, time of ovulation, endogenous estradiol 17beta and luteinizing hormone in Bos Indicus cows after a synchronization program. Reprod Dom Anim.

